# Supplementary material for: A Likelihood Approach for Real-Time Calibration of Stochastic Compartmental Epidemic Models
Source: PLoS Comput Biol. 2017 Jan 17;13(1):e1005257. doi: 10.1371/journal.pcbi.1005257 (PMC5240920; doi:10.1371/journal.pcbi.1005257)
Supplement: S1 File — (TAR.GZ) [file pcbi.1005257.s014.tar.gz › HSPH_Online-SI-Revision/EnKF07/n10-severe/n10-severe_EnKF_table-4.pdf]

1	R0	{1.68952, 11.8402}	{2.34565, 6.80618}	{{0.669776, 0.329377}, {3., 10.1513}}	{70.1745, 42.5164}	inf	719.	839.912	{42.3187, 3794.5}	145.516	0.787346	newly	473.	925.84	{21., 3526.}	196.753	0.756814	Reff	1.34131	0.598582	{0.0697795, 0.962905}	66.2983	ar	6872.	18 233.2	{15 764., 25 199.}	165.326
2	R0	{1.71739, 9.62661}	{2.50443, 7.08965}	{{1.04166, 1.48507}, {3., 19.614}}	{57.3551, 85.4762}	inf	504.	356.808	{50.1106, 499.591}	80.5881	0.525497	newly	426.	927.23	{52., 1237.}	216.19	0.589003	Reff	1.47352	0.637524	{0.221987, 0.952971}	64.5113	ar	6874.	18 148.7	{16 573., 18 688.}	164.019
3	R0	{1.48936, 6.23975}	{2.40465, 5.17166}	{{1.46474, 2.34461}, {3., 9.95202}}	{62.2892, 59.8514}	inf	371.	420.677	{15.0695, 1050.1}	112.125	0.582959	newly	491.	832.31	{18., 4204.}	178.894	0.720618	Reff	1.29008	0.703717	{0.0986792, 1.46641}	56.7457	ar	5666.	19 108.1	{16 876., 25 215.}	237.241
4	R0	{1.62394, 7.51135}	{2.71934, 12.8412}	{{2.32188, 6.8182}, {3., 16.4224}}	{67.4531, 77.7858}	inf	401.	482.556	{134.739, 337.079}	69.5799	0.207068	newly	435.	242.6	{51., 344.}	66.377	0.461274	Reff	1.42192	0.531976	{0.263183, 0.693746}	64.685	ar	6417.	17 070.4	{15 732., 18 355.}	166.019
5	R0	{1.53632, 8.57652}	{2.7155, 12.0208}	{{2.31482, 6.74484}, {3., 15.5797}}	{76.7536, 57.2446}	inf	485.	428.901	{173.99, 552.848}	60.6574	0.255208	newly	460.	233.55	{67., 283.}	65.463	0.450889	Reff	1.29543	0.546822	{0.344085, 0.751916}	60.8267	ar	6260.	16 907.2	{15 605., 18 178.}	170.083
6	R0	{1.47166, 5.7434}	{2.30044, 6.93847}	{{0.704481, 0.0507143}, {3., 11.539}}	{85.249, 99.9054}	inf	232.	627.431	{19.6405, 1128.96}	226.975	0.537095	newly	328.	847.14	{12., 1447.}	235.03	0.70447	Reff	1.32906	0.578413	{0.060702, 0.838928}	66.2985	ar	5650.	17 703.9	{15 753., 22 145.}	213.343
7	R0	{1.48179, 8.87369}	{2.70324, 13.1207}	{{2.29962, 6.8713}, {3., 17.4943}}	{82.4305, 66.3648}	inf	510.	458.787	{305.063, 374.183}	63.8821	0.264908	newly	397.	225.64	{99., 372.}	58.8262	0.38745	Reff	1.14379	0.556176	{0.251401, 0.838729}	58.3168	ar	5233.	16 871.1	{15 566., 18 849.}	222.397
8	R0	{1.53669, 6.47807}	{2.12337, 9.82012}	{{0.425428, 5.70689}, {3., 14.2433}}	{75.0381, 63.7327}	inf	392.	400.413	{45.8955, 306.99}	77.9463	0.394419	newly	477.	287.01	{20., 329.}	79.9979	0.65789	Reff	1.32417	0.416744	{0.055757, 0.657011}	69.0404	ar	5934.	17 908.1	{16 844., 19 990.}	201.787
9	R0	{1.57227, 6.20989}	{2.15885, 3.97069}	{{1.14594, 2.67987}, {3., 5.15348}}	{58.4794, 40.5256}	inf	215.	1488.82	{403.195, 3111.71}	592.668	0.696437	newly	311.	1698.79	{681., 4547.}	447.412	0.591071	Reff	1.49381	0.417763	{0.0802674, 1.18505}	72.178	ar	6136.	15 221.1	{6153., 20 598.}	151.096
10	R0	{1.54936, 6.50938}	{2.17824, 4.31992}	{{0.963121, 2.68287}, {3., 5.18946}}	{63.3801, 40.383}	inf	423.	1434.32	{574.124, 3226.93}	243.792	0.437677	newly	555.	1652.97	{551., 4311.}	205.915	0.360273	Reff	1.36483	0.429332	{0.0831317, 0.899363}	68.8082	ar	6275.	15 850.9	{8004., 20 697.}	157.181
11	R0	{1.42179, 3.18601}	{1.97201, 3.63973}	{{0.152199, 0.859344}, {3., 6.02693}}	{84.1037, 80.4819}	inf	124.	643.376	{119.11, 1404.73}	424.796	0.533346	newly	375.	817.23	{91., 1712.}	164.291	0.408278	Reff	1.33549	0.331592	{0.0250177, 0.459271}	76.0678	ar	5322.	16 615.8	{14 160., 19 776.}	212.21
12	R0	{1.48035, 6.46183}	{2.03078, 3.69079}	{{0.144759, 0.863412}, {3., 5.99065}}	{82.2791, 52.2396}	inf	191.	498.294	{139.371, 1197.58}	174.607	0.365271	newly	266.	601.73	{103., 1276.}	159.229	0.38604	Reff	1.39049	0.314689	{0.0192429, 0.460594}	77.3685	ar	5793.	16 869.5	{15 112., 18 729.}	191.205
13	R0	{1.75755, 8.53854}	{2.09968, 9.18735}	{{0.43914, 5.81926}, {3., 14.3669}}	{62.698, 45.9581}	inf	456.	399.608	{28.2218, 311.034}	80.8933	0.489663	newly	444.	323.16	{13., 402.}	86.1667	0.669888	Reff	1.57195	0.392895	{0.048268, 0.65932}	75.0059	ar	6832.	17 815.9	{16 832., 21 546.}	160.772
14	R0	{1.47355, 4.89451}	{2.48456, 5.38919}	{{2.01911, 4.19409}, {3., 7.66514}}	{73.6334, 29.1826}	inf	339.	256.187	{105.408, 367.103}	88.4915	0.493842	newly	540.	330.73	{69., 496.}	92.2019	0.655085	Reff	1.31367	0.565911	{0.175947, 1.42086}	68.3703	ar	5710.	18 334.	{16 635., 24 257.}	221.086
15	R0	{1.62071, 5.98665}	{2.48722, 7.2724}	{{2.14521, 3.58776}, {3., 14.6816}}	{54.913, 50.8775}	inf	269.	2144.84	{564.597, 3820.64}	704.375	0.808206	newly	451.	2540.17	{567., 7217.}	474.024	0.620566	Reff	1.51245	1.03261	{0.391209, 2.20295}	51.7054	ar	6635.	15 193.3	{10 141., 22 065.}	130.167
16	R0	{1.67268, 7.7256}	{2.52201, 5.56152}	{{2.00827, 4.1439}, {3., 7.65912}}	{53.7877, 28.0266}	inf	657.	229.592	{32.9462, 345.352}	80.3739	0.742791	newly	678.	256.54	{25., 520.}	77.0737	0.744385	Reff	1.34935	0.595559	{0.176462, 1.70144}	67.2792	ar	6947.	17 860.4	{16 414., 22 583.}	158.012
17	R0	{1.67805, 11.1417}	{2.46711, 5.42978}	{{1.24631, 4.19296}, {3., 7.77314}}	{54.3326, 51.2662}	inf	455.	157.789	{23.6411, 270.337}	74.0446	0.649291	newly	326.	177.59	{15., 414.}	69.9049	0.5638	Reff	1.48021	0.48509	{0.157184, 1.08514}	71.5741	ar	6625.	17 648.3	{16 553., 19 291.}	166.957
18	R0	{1.66743, 9.99046}	{2.08981, 9.1611}	{{0.425982, 5.72958}, {3., 14.4326}}	{67.7353, 42.8779}	inf	487.	382.982	{29.4185, 301.554}	79.867	0.53312	newly	415.	306.69	{14., 377.}	86.2386	0.682489	Reff	1.42932	0.388637	{0.0473135, 0.667316}	72.8097	ar	6899.	17 872.3	{16 908., 21 170.}	159.056
19	R0	{1.70787, 9.85478}	{2.76864, 11.9912}	{{2.31473, 6.74803}, {3., 15.6645}}	{62.1108, 47.5299}	inf	650.	447.802	{296.183, 296.183}	70.3354	0.360392	newly	499.	248.42	{100., 293.}	71.7796	0.483584	Reff	1.37449	0.591729	{0.275452, 1.13821}	62.4709	ar	6867.	17 165.4	{15 746., 21 256.}	149.969
20	R0	{1.53809, 10.5848}	{2.7574, 10.871}	{{2.31988, 6.53598}, {3., 13.5479}}	{79.2745, 31.775}	inf	420.	349.972	{86.9422, 350.562}	59.3854	0.332609	newly	300.	224.03	{34., 323.}	57.6767	0.413753	Reff	1.34152	0.565561	{0.221427, 0.937657}	64.1606	ar	6108.	16 977.9	{15 401., 20 998.}	177.962
21	R0	{1.59925, 6.74479}	{2.10453, 4.61992}	{{0.971899, 2.68239}, {3., 8.79521}}	{56.788, 43.375}	inf	387.	1266.44	{335.317, 2422.05}	237.55	0.440981	newly	488.	1413.28	{334., 3317.}	203.668	0.37962	Reff	1.42989	0.428377	{0.084245, 1.06507}	70.4064	ar	6411.	14 544.6	{6994., 19 516.}	133.64
22	R0	{1.38779, 4.40977}	{2.43872, 5.66698}	{{1.21835, 4.21631}, {3., 7.84369}}	{79.6615, 34.282}	inf	183.	259.741	{17.7582, 343.971}	119.07	0.403534	newly	337.	278.51	{10., 497.}	101.101	0.641198	Reff	1.26761	0.460257	{0.165172, 1.05551}	70.547	ar	5154.	17 963.2	{16 599., 19 755.}	248.529
23	R0	{1.53657, 7.50352}	{2.41283, 5.40181}	{{1.46381, 2.31112}, {3., 9.9188}}	{59.5344, 53.1224}	inf	441.	352.023	{15.1319, 740.516}	94.9794	0.615634	newly	429.	670.7	{15., 2904.}	163.869	0.678342	Reff	1.31868	0.617531	{0.0458629, 1.43645}	60.2586	ar	5928.	18 462.5	{16 827., 23 426.}	211.446
24	R0	{1.71131, 6.24526}	{2.45963, 5.93472}	{{1.27001, 4.22496}, {3., 7.84826}}	{50.1736, 28.9061}	inf	217.	225.714	{15.2789, 278.097}	93.7476	0.458422	newly	358.	238.27	{11., 472.}	86.6676	0.665076	Reff	1.60743	0.514738	{0.165793, 1.11465}	71.1064	ar	6924.	17 863.1	{16 494., 19 383.}	157.988
25	R0	{1.4412, 3.07729}	{2.32299, 5.07143}	{{1.68098, 1.2079}, {3., 13.1539}}	{64.1624, 106.403}	inf	56.	2585.62	{498.035, 5481.41}	4518.33	1.48748	newly	207.	2975.14	{472., 7974.}	1340.02	0.968044	Reff	1.41454	0.63452	{0.172852, 1.34408}	56.8085	ar	5810.	15 857.1	{7184., 25 635.}	174.964
26	R0	{1.4159, 4.93858}	{2.21141, 4.49361}	{{0.886751, 2.68155}, {3., 5.13354}}	{73.7265, 31.0208}	inf	225.	1428.39	{383.129, 3207.8}	537.108	0.665277	newly	397.	1493.73	{367., 4006.}	283.458	0.451079	Reff	1.30079	0.36159	{0.114151, 0.586418}	73.907	ar	5255.	15 523.	{8369., 21 354.}	198.986
27	R0	{1.57465, 7.05414}	{2.5392, 5.47163}	{{1.82171, 0.703262}, {3., 13.1475}}	{62.6347, 63.1333}	inf	216.	2296.05	{512.553, 5362.78}	963.825	0.902687	newly	292.	2553.87	{527., 6482.}	776.455	0.773945	Reff	1.4934	0.587984	{0.16012, 1.06716}	62.2415	ar	6359.	14 873.9	{9338., 23 578.}	133.904
28	R0	{1.41422, 4.62402}	{2.11969, 3.96134}	{{0.875569, 2.67892}, {3., 5.1489}}	{68.7494, 31.9048}	inf	138.	1394.22	{284.309, 3682.34}	911.847	0.838814	newly	285.	1478.91	{380., 4147.}	424.453	0.584084	Reff	1.35298	0.370489	{0.107472, 0.727981}	72.6169	ar	5485.	15 236.7	{8109., 19 688.}	181.875
29	R0	{1.41901, 3.63017}	{2.48884, 7.49872}	{{2.12919, 3.66375}, {3., 14.691}}	{78.1223, 108.775}	inf	127.	2172.5	{515.928, 4475.41}	1616.32	1.09786	newly	382.	2301.22	{320., 6902.}	515.204	0.655598	Reff	1.3631	0.91399	{0.304725, 2.15323}	53.3613	ar	5979.	15 108.3	{9038., 22 245.}	153.569
30	R0	{1.60719, 4.72984}	{2.55232, 7.20621}	{{2.12913, 3.852}, {3., 14.694}}	{60.4368, 65.1703}	inf	120.	2179.47	{646.887, 4261.93}	1719.23	1.14847	newly	274.	2286.33	{615., 5800.}	739.96	0.790028	Reff	1.56171	0.882746	{0.298826, 1.80621}	52.8117	ar	6479.	14 516.2	{8632., 21 241.}	125.175
31	R0	{1.38175, 6.26468}	{2.17589, 3.96116}	{{0.119376, 0.743119}, {3., 5.8998}}	{94.1119, 53.1045}	inf	357.	567.554	{140.944, 1155.12}	96.5535	0.278505	newly	432.	669.12	{123., 1175.}	106.454	0.34036	Reff	1.2035	0.326135	{0.0213213, 0.460806}	74.9936	ar	5397.	16 384.7	{14 947., 18 188.}	205.152
32	R0	{1.4947, 8.8847}	{2.71307, 12.5122}	{{2.30819, 6.78827}, {3., 16.3586}}	{81.5129, 59.7051}	inf	462.	517.333	{281.428, 281.428}	86.6985	0.279797	newly	361.	256.08	{95., 287.}	70.7479	0.394912	Reff	1.18634	0.572905	{0.257904, 0.856226}	56.3965	ar	5407.	17 141.	{15 745., 18 882.}	217.015
33	R0	{1.55891, 8.28978}	{2.72055, 11.1046}	{{2.31157, 4.21271}, {3., 15.0702}}	{74.5159, 63.4515}	inf	446.	786.108	{167.931, 1435.21}	105.019	0.223051	newly	409.	569.78	{95., 609.}	112.22	0.373614	Reff	1.30107	0.63206	{0.252825, 1.18871}	57.4026	ar	6239.	17 937.	{15 850., 24 661.}	187.497
34	R0	{1.60764, 10.4729}	{2.75118, 13.0585}	{{2.30383, 5.90794}, {3., 16.8027}}	{71.1316, 48.7192}	inf	658.	717.186	{195.155, 1608.54}	88.6467	0.338207	newly	455.	372.8	{98., 611.}	81.767	0.424984	Reff	1.23097	0.545168	{0.19319, 0.820391}	60.4891	ar	6495.	17 391.2	{15 747., 19 205.}	167.763
35	R0	{1.48527, 5.74485}	{1.9693, 3.7116}	{{0.13605, 0.845182}, {3., 6.01096}}	{79.2655, 47.6358}	inf	223.	648.04	{126.063, 1407.21}	210.556	0.376927	newly	365.	752.49	{99., 1751.}	149.592	0.391904	Reff	1.37566	0.322608	{0.0212891, 0.461179}	76.5488	ar	5811.	16 635.9	{14 605., 19 881.}	187.7
36	R0	{1.61457, 9.93364}	{2.54127, 9.19225}	{{1.11766, 1.58824}, {3., 19.4951}}	{66.5394, 86.997}	inf	466.	361.12	{47.094, 847.03}	85.9675	0.560283	newly	363.	838.85	{44., 1175.}	241.672	0.6751	Reff	1.39273	0.638989	{0.232958, 0.915206}	64.3836	ar	6338.	18 187.2	{16 481., 21 643.}	186.955
37	R0	{1.47831, 7.08139}	{2.45536, 5.46463}	{{2.01738, 4.18243}, {3., 7.76301}}	{71.1636, 29.6972}	inf	307.	156.055	{13.6881, 263.684}	67.1371	0.512482	newly	351.	174.89	{11., 373.}	70.0427	0.588883	Reff	1.31939	0.488236	{0.175162, 1.03898}	68.816	ar	5530.	17 435.4	{16 517., 18 949.}	215.787
38	R0	{1.73445, 9.8858}	{2.47472, 5.50376}	{{2.01834, 4.2008}, {3., 7.78489}}	{49.8586, 44.3266}	inf	459.	160.053	{23.5472, 269.074}	73.5985	0.644811	newly	402.	183.32	{15., 434.}	69.4179	0.604144	Reff	1.53534	0.507271	{0.15943, 1.07398}	71.3628	ar	6842.	17 901.	{16 529., 20 234.}	161.634
39	R0	{1.66621, 11.4824}	{2.53249, 6.78389}	{{1.6726, 3.53507}, {3., 9.13648}}	{51.9908, 40.9193}	inf	585.	485.628	{131.072, 1014.35}	90.6405	0.511027	newly	373.	525.01	{117., 722.}	125.182	0.433452	Reff	1.36629	0.58193	{0.205189, 0.79236}	63.9405	ar	6473.	18 463.	{16 331., 26 360.}	185.231
40	R0	{1.60694, 9.95741}	{2.53485, 4.84431}	{{1.6772, 1.16587}, {3., 6.39158}}	{57.7439, 51.3497}	inf	536.	433.971	{222.864, 778.283}	82.1626	0.410261	newly	419.	525.93	{167., 751.}	100.126	0.334373	Reff	1.35481	0.577201	{0.187775, 0.793813}	63.7508	ar	6625.	18 440.6	{16 419., 25 838.}	178.348
41	R0	{1.55128, 4.71911}	{2.53097, 7.69144}	{{2.13077, 3.86511}, {3., 14.7079}}	{65.4813, 73.1672}	inf	143.	2076.79	{649.732, 3332.23}	1355.59	1.0619	newly	305.	2191.74	{568., 6961.}	624.872	0.717317	Reff	1.48892	0.888042	{0.296383, 2.07068}	52.591	ar	6141.	14 515.3	{8947., 22 205.}	137.362
42	R0	{1.60063, 6.81118}	{2.28238, 10.0403}	{{0.694427, 6.90498}, {3., 12.3288}}	{75.1812, 47.4084}	inf	336.	752.039	{21.5107, 1710.42}	181.041	0.532151	newly	454.	529.17	{10., 864.}	120.43	0.733524	Reff	1.44265	0.59036	{0.0975023, 0.839217}	67.2963	ar	6528.	18 024.6	{15 850., 21 116.}	176.112
43	R0	{1.56986, 5.44652}	{2.46378, 4.71676}	{{1.72005, 0.38183}, {3., 12.8065}}	{57.5134, 69.3128}	inf	263.	2359.63	{511.197, 5514.76}	802.732	0.837227	newly	451.	2800.49	{435., 7445.}	531.284	0.665894	Reff	1.45887	0.555853	{0.188367, 1.06036}	63.3726	ar	6312.	15 534.5	{9025., 24 590.}	147.562
44	R0	{1.47523, 8.07785}	{2.52212, 9.57028}	{{1.11933, 1.84449}, {3., 19.4698}}	{79.1166, 104.227}	inf	404.	380.233	{61.502, 839.204}	84.4406	0.431452	newly	403.	822.88	{47., 1194.}	212.412	0.618126	Reff	1.27976	0.663735	{0.246006, 0.99158}	61.0566	ar	5692.	18 218.9	{16 523., 22 007.}	220.078
45	R0	{1.6417, 7.69429}	{2.53165, 5.49243}	{{2.01782, 4.17863}, {3., 7.7046}}	{60.2334, 28.7502}	inf	406.	228.	{58.7922, 268.978}	81.8783	0.551944	newly	444.	321.86	{36., 501.}	96.4189	0.604818	Reff	1.44486	0.542817	{0.156879, 1.3334}	70.4728	ar	6735.	17 769.4	{16 535., 20 657.}	165.767
46	R0	{1.38865, 5.83473}	{2.47087, 5.20001}	{{1.47939, 2.37951}, {3., 10.0341}}	{77.933, 63.1308}	inf	250.	331.317	{37.2666, 1044.18}	94.0123	0.331017	newly	337.	682.13	{26., 4178.}	176.881	0.459775	Reff	1.23465	0.587731	{0.0420382, 1.46375}	59.5188	ar	5233.	18 454.9	{16 888., 25 147.}	252.664
47	R0	{1.45289, 6.87903}	{2.4371, 5.44166}	{{2.01411, 4.17151}, {3., 7.75355}}	{72.7373, 30.0553}	inf	272.	155.193	{12.3048, 266.307}	66.7125	0.492158	newly	303.	172.79	{10., 401.}	70.868	0.564939	Reff	1.28537	0.484875	{0.161577, 1.02335}	68.5678	ar	5379.	17 225.1	{16 441., 18 862.}	221.701
48	R0	{1.69416, 9.4831}	{2.49963, 10.4682}	{{1.04112, 3.65688}, {3., 19.6593}}	{58.384, 65.0369}	inf	453.	370.945	{44.1818, 309.96}	85.2553	0.477961	newly	442.	573.58	{42., 400.}	160.846	0.6682	Reff	1.51407	0.598305	{0.139137, 0.854186}	67.665	ar	6925.	18 007.5	{16 502., 18 343.}	160.037
49	R0	{1.47843, 7.16286}	{2.4297, 5.55073}	{{1.46683, 2.36299}, {3., 10.0474}}	{64.6705, 54.9306}	inf	357.	378.11	{16.4391, 1043.91}	104.732	0.547696	newly	403.	745.37	{16., 4170.}	187.923	0.657516	Reff	1.2988	0.634872	{0.0409996, 1.45559}	59.1697	ar	5449.	18 664.3	{16 862., 24 930.}	242.527
50	R0	{1.68843, 5.72427}	{2.55772, 6.32705}	{{2.14511, 3.49161}, {3., 14.5913}}	{53.2061, 43.4426}	inf	329.	2117.93	{675.938, 3552.22}	548.158	0.725124	newly	521.	2703.44	{644., 7231.}	425.624	0.581653	Reff	1.53732	0.973244	{0.302768, 2.20478}	53.3437	ar	6800.	14 978.7	{8603., 22 483.}	122.001
